# Molecular Features and Antimicrobial Susceptibilities of *Streptococcus equi* ssp. *equi* Isolates from Strangles Cases in Indonesia

**DOI:** 10.3390/vetsci10010049

**Published:** 2023-01-10

**Authors:** Dordia Anindita Rotinsulu, Christa Ewers, Katharina Kerner, Amrozi Amrozi, Retno Damayanti Soejoedono, Torsten Semmler, Rolf Bauerfeind

**Affiliations:** 1 Institute for Hygiene and Infectious Diseases of Animals, Justus Liebig University Giessen, 35392 Giessen, Germany; 2 School of Veterinary Medicine and Biomedical Sciences, IPB University, Bogor 16680, Indonesia; 3 NG-1 Microbial Genomics, Robert Koch Institute, 13353 Berlin, Germany

**Keywords:** strangles, *Streptococcus equi*, antimicrobial susceptibility, cgMLST, *seM* typing, MLST, whole genome sequencing

## Abstract

**Simple Summary:**

Strangles is a highly infectious disease of equines caused by *Streptococcus equi* ssp. *equi (S. equi equi)*. The objective of this study was to characterize *S. equi equi* isolates obtained from suspected strangles cases in Indonesia using whole genome sequence-based analyses and antimicrobial susceptibility testing. Isolates were recovered from seven diseased horses on four farms in three different provinces in 2018. All these *S. equi equi* isolates were classified as ST179 and carried *seM* allele 166. Isolates differed from each other by only 2 to 14 core genome (cg) SNPs and built an exclusive sub-cluster in Bayesian Analysis of Population Structure cluster 2 (BAPS-2) of the cgMLST scheme. All isolates revealed predicted amino acid sequence identity to seven and high similarity to one of the eight antigen fragments contained in the Strangvac^®^ recombinant subunit vaccine. Furthermore, all isolates were susceptible to beta-lactam antibiotics penicillin G, ampicillin, and ceftiofur. Our data suggest that the horses referred to in this study were affected by strains of the same novel sublineage within globally distributed BAPS-2 of *S. equi equi*. Nevertheless, penicillin G can be used as a first-choice antibiotic for treatment and Strangvac^®^ may also be protective against these strains.

**Abstract:**

Strangles, caused by *Streptococcus equi* ssp. *equi* (*S. equi equi*), is a highly infectious and frequent disease of equines worldwide. No data are available regarding the molecular epidemiology of strangles in Indonesia. This study aimed to characterize *S. equi equi* isolates obtained from suspected strangles cases in Indonesia in 2018. Isolates originated from seven diseased horses on four different farms located in three provinces of Indonesia. Whole genome sequences of these isolates were determined and used for *seM* typing, multilocus sequence typing (MLST), and core genome MLS typing (cgMLST). Genomes were also screened for known antimicrobial resistance genes and genes encoding for the recombinant antigens used in the commercial Strangvac^®^ subunit vaccine. All seven *S. equi equi* isolates from Indonesia belonged to ST179 and carried *seM* allele 166. Isolates differed from each other by only 2 to 14 cgSNPs and built an exclusive sub-cluster within the Bayesian Analysis of Population Structure (BAPS) cluster 2 (BAPS-2) of the *S. equi equi* cgMLST scheme. All isolates revealed predicted amino acid sequence identity to seven and high similarity to one of the eight antigen fragments contained in Strangvac^®^. Furthermore, all isolates were susceptible to beta-lactam antibiotics penicillin G, ampicillin, and ceftiofur. Our data suggest that the horses from this study were affected by strains of the same novel sublineage within globally distributed BAPS-2 of *S. equi equi*. Nevertheless, penicillin G can be used as a first-choice antibiotic against these strains and Strangvac^®^ may also be protective against Indonesian strains.

## 1. Introduction

Strangles is a highly infectious disease of equines that is caused by *Streptococcus equi* subspecies *equi* (*S. equi equi*) [1,2]. Morbidity and mortality rates of strangles may be up to 100% [3,4] and 10% [5], respectively, causing a significant economic loss and animal welfare burden [6]. As a result of prolonged recovery periods, movement restrictions, and associated serious complications, losses may exceed £250,000 per outbreak [7]. An important factor in the epidemiology of strangles is the persistence of *S. equi equi* for up to several years in subclinical carriers that may become the unrecognized source of infection for other equines [8,9,10]. While strangles was recorded in the year 1251 for the first time [11], treatment and control of strangles still pose a challenge [1,10]. Antimicrobials, particularly beta-lactams, are routinely used to treat affected horses but can have also adverse effects on the course of infection depending on the stage, manifestation, and severity of disease [1,12]. Favorably, resistance against antibiotics is rare in *S. equi equi*, except resistance against aminoglycosides [13,14], trimethoprim/sulfamethoxazole [13,14,15], and fluoroquinolones [14]. For immunoprophylaxis, several types of *S. equi equi* vaccines have been developed and used with varying degrees of success, such as cell-free protein extract vaccines [16] and live attenuated vaccines [1,9]. The latest development is a recombinant subunit vaccine named Strangvac^®^ [17] that received marketing authorisation in the European Union in August 2021 [18].

*Streptococcus equi* subspecies *equi* is a Gram-positive, beta-haemolytic *Streptococcus* classified in Lancefield group C [19]. It shares 97% DNA sequence homology with *S. equi* subspecies *zooepidemicus* (*S. equi zooepidemicus*) [20]. In recent years, several DNA sequence-based methods have been implemented not only to unravel the phylogeny of this pathogen but also to trace disease outbreaks among equines more precisely. A single locus sequence typing (SLST) method, known as *seM* typing, has been used for *S. equi equi* as an epidemiological tool to investigate strangles outbreaks [21,22,23]. The *seM* gene encodes the *S. equi equi* cell wall-associated M-protein (SeM) that has an antiphagocytic activity by binding fibrinogen and immunoglobulin G [24]. Furthermore, a multilocus sequence typing (MLST) scheme for *S. equi equi* and *S. equi zooepidemicus* defines sequence types (STs) based on the allelic profile presented by internal sections of seven conserved housekeeping genes [25]. Moreover, a novel core genome MLST (cgMLST) scheme for *S. equi equi* enables molecular characterization and phylogenomic analysis in more detail [2].

Strangles has been reported from equines worldwide, except for Iceland [26], but incidence and prevalence data are generally scarce in the literature. At least for the United Kingdom, the strangles incidence was estimated at more than 600 outbreaks per year [27]. In Indonesia, strangles occurs sporadically [28,29] and since 2018, it is listed as a notifiable equine disease [30]. However, no data have been published yet regarding molecular features, antigens or antimicrobial susceptibility patterns of *S. equi equi* strains from Indonesia. Therefore, this study aimed to determine molecular features of *S. equi equi* isolates obtained from strangles cases in Indonesia based on whole genome sequence (WGS) data analyses and to provide antimicrobial susceptibility data. In addition, we assessed the phylogenetic relationship of the Indonesian isolates to 759 other *S. equi equi* strains that had been recovered from horses worldwide and whose genome data were publicly available [2,31,32,33,34]. We also conducted an in silico analysis to examine amino acid similarities between the eight antigen fragments contained in Strangvac^®^ and their homologues encoded by the *S. equi equi* isolates from Indonesia. Furthermore, in vitro susceptibilities of Indonesian *S. equi equi* isolates were examined to 15 antimicrobials, including beta-lactam antibiotics such as penicillin G, ampicillin, ceftiofur, and cephalothin.

## 2. Materials and Methods

### 2.1. Bacterial Isolates and Reference Strains

We examined *S. equi equi* isolates from seven horses suspected of suffering from strangles on four different farms in three provinces (West, Central, and East Java) in Indonesia during 2018 (Table 1). Field veterinarians collected samples of nasal discharges (horses 2, 3, 5, 6, and 7) and aspirates from abscessing mandibular lymph nodes (horses 1 and 4) of the diseased horses. Subsequently, the samples were submitted at ambient temperature to the Division of Medical Microbiology, School of Veterinary Medicine and Biomedical Sciences, IPB University, Bogor, Indonesia. All samples were systematically examined for *S. equi equi* by standard bacterial culture methods. Briefly, specimens were inoculated onto 5% (*v*/*v*) sheep blood agar plates (BAP) and cultured at 37 °C for 24 and 48 h of incubation. Single colonies of beta-haemolytic, Gram-positive cocci were then subcultured onto BAP and incubated for 24 h prior to further examination. Clinical and geographical data associated with the isolates were recorded as supplied by the veterinarians.

Based on colony character, cell morphology, and biochemical reactions, one putative *S. equi equi* isolate was saved from each horse and shipped to the Institute for Hygiene and Infectious Diseases of Animals, Justus Liebig University Giessen, Germany, for further examination. Reference strains *S. equi equi* DSM 20561 (synonym: ATCC 33398, NCDO 2493, NCTC 9682) and *S. equi zooepidemicus* DSM 20727 (synonym: ATCC 43079, NCDO 1358, NCTC 7023) were purchased from the Leibniz Institute DSMZ (German Collection of Microorganisms and Cell Cultures GmbH, Braunschweig, Germany).

### 2.2. Taxonomic Assessment of Bacterial Isolates

Bacterial isolates were identified to the species and subspecies level by assessment of growth characteristics on BAP, cell morphology including Gram staining, biochemical reactions, and presence of marker DNA sequences, as well as by Matrix Assisted Laser Desorption Ionization Time of Flight mass spectrometry (MALDI-TOF MS). Biochemical reactions and enzymatic activity were tested using API^®^ RAPID ID 32 STREP (bioMérieux, Marcy l’Etoile, France) according to the manufacturer’s manual. All isolates were re-assessed by MALDI-TOF MS with the Microflex LT MALDI-TOF mass spectrometer V.3.3.1.0 (MBT 7854 MSP library; Bruker Daltonics, Bremen, Germany). To do this, a bacterial colony was selected from the BAP and applied to a spot on the target plate directly (direct transfer) or after acetonitrile-formic acid extraction (extended direct transfer) according to the manufacturer’s instructions. The resulting spectra were compared against reference spectra (MBT 7854 MSP library, Bruker Daltonics). A multiplex polymerase chain reaction (PCR) was used to test bacterial isolates for the presence of the ICE*Se*2 locus of *S. equi equi*, the ICE*Sz*1 locus of *S. equi zooepidemicus*, and the *sodA* gene that typically is encoded by both *S. equi* subspecies [35]. Briefly, the bacteria in question were suspended in sterile distilled water (200 μL) and boiled for 10 min, followed by centrifugation at 17,000× *g* for 5 min at ambient temperature using Centrifuge 5804R (Eppendorf, Hamburg, Germany). The supernatant was used as PCR template. The primer set was compiled as described by others [35]. The PCR mix (30 μL) consisted of 3 µL primer mix (each primer 5 µM: sodAGC1 F and R, ICESz1GC5 F and R, ICESE2GC2 F and R), 3 μL 10 × DreamTaq Green Buffer (Thermo Fisher Scientific, Waltham, Massachusetts, USA), 1 μL 10 mM dNTPs (Rapidozym Gmbh, Berlin, Germany), 0.2 μL DreamTaq DNA Polymerase (Thermo Fisher Scientific), 19.8 μL sterile deionised water and 3 μL of the template. PCR was performed on a Biometra TRIO 48 thermal cycler (Analytik Jena, Jena, Germany) using the following protocol: 94 °C for 5 min (initial denaturation), 35 cycles at 94 °C for 30 s, 54 °C for 30 s, and 72 °C for 60 s (amplification), and 72 °C for 5 min (final elongation). In each PCR run, reference strains of *S. equi equi* and *S. equi zooepidemicus*, as well as sterile deionised water were used as positive and negative controls, respectively. The presence of desired amplicons (201 bp, 158 bp, and 380 bp, respectively) was examined by UV illumination of PCR products after electrophoresis through 2% agarose gels containing ethidium bromide.

### 2.3. Bacterial DNA Extraction and Whole Genome Sequencing

One *S. equi equi* isolate from each horse (n = 7) was whole genome sequenced. DNA was isolated from bacteria according to a published phenol-chloroform extraction method [36] with the following modifications. Bacteria were inoculated into 15 mL brain heart infusion broth (Thermo Fisher Scientific) and incubated at 37 °C overnight, followed by centrifugation for 10 min at 4500× *g* at ambient temperature using Eppendorf Centrifuge 5804R. The pellet was suspended in 200 µL TES buffer (50 mM Tris-HCl, 5 mM EDTA, 50 mM NaCl, pH 8.5) and 15 µL of lysozyme (10 mg/mL) and incubated for 30 min at 37 °C, followed by addition of 10 µL of 10% sodium dodecyl sulfate (SDS), and 10 µL proteinase K (20 mg/mL). After incubation at 55 °C for 60 min, 12 µL of RNAse (500 µg/mL) (Roche, Basel, Switzerland) was added prior to incubation at 37 °C for 30 min. The solution was extracted three times with phenol-chloroform, precipitated with ethanol, and finally solubilized in elution buffer (10 mM Tris-Cl 9, pH 8.5).

Whole genome sequence libraries were generated with the Nextera XT DNA Library Preparation Kit (Illumina, San Diego, California, USA) following the manufacturer’s instructions at the Robert Koch Institute, Berlin, Germany. The sequencing was carried out on a MiSeq sequencer (MiSeq Reagent Kit V.3; Illumina Inc., San Diego, CA), resulting in paired-end reads of 300 bp with an average coverage of 90×. Adapter trimming was performed by Flexbar on Illumina paired-end reads [37], while BayesHammer was used to correct the reads [38]. The de novo assembly was performed using SPAdes V.3.11.1 [39] with default settings. Afterwards, all genomes were annotated with Prokka V.1.13 [40].

### 2.4. SeM Typing

For the *seM* typing, an internal fragment of 327 bp near the N-terminal domain of the *seM* gene was analysed [21]. *SeM* alleles and SeM peptides were assigned using the PubMLST database (http://pubmlst.org/szooepidemicus/, accessed on 30 September 2022) [41]. A neighbor-joining tree of the *seM* variable region was constructed using Geneious V.8.1.9 (Biomatters Ltd., Auckland, New Zealand) in order to compare the *seM* allele found in this study with alleles deposited in the public PubMLST database.

### 2.5. MLST

Internal fragments of seven housekeeping genes of *S. equi equi* were analysed for MLST, namely carbamate kinase (*arcC*), ribonucleoside-diphosphate reductase (*nrdE*), prolyl-tRNA synthetase (*proS*), signal peptidase I (*spi*), thymidylate kinase (*tdk*), triosephosphate isomerase (*tpi*), and acetyl-CoA acetyltransferase (*yqiL*) [25]. The STs were assigned using the PubMLST database (http://pubmlst.org/szooepidemicus/, accessed on 30 September 2022) [41].

### 2.6. The cgMLST Analysis

The cgMLST scheme comprises 1286 core genes from the *S. equi equi* reference strain Se4047 (GenBank accession number: FM204883.1) by excluding the mobile genetic elements, insertion sequences, sortase-processed protein genes, and repeats (*hasC1*, *hasC2*) [2]. Se4047 had been isolated from a pony with strangles in the New Forest, England, in 1990 [20,25]. Alleles were assigned based on variations to the Se4047 core genome. Furthermore, genomes of *S. equi equi* in this study were compared with 759 publicly available genomes of *S. equi equi* that were retrieved from Pathogenwatch [2,31,32,33,34]. The pairwise score matrix (excluding indels) was used to construct the dendrogram using the Analysis of Phylogenetics and Evolution (APE) package [42]. Phylogentic reconstruction of the genomes of this study (n = 7) and publicly available genomes (n = 759) were generated in Pathogenwatch (https://pathogen.watch/, accessed on 30 September 2022) as described before [2]. The genomes and data can be accessed at https://pathogen.watch/collection/pi6lq3wwlwkw-paper-2022-see-indonesia-766-genomes (accessed on 30 December 2022). Metadata and phylogenetic reconstruction were visualized using Microreact [43] and can be accessed at https://microreact.org/project/bW2dCd66hx3A9wxbGT1rW4-s-equi-equi-indonesia-7-and-worldwide-759 (accessed on 30 December 2022).

### 2.7. Assessment of Antigens Used in the Strangvac^®^ Vaccine

Reference DNA sequences encoding for the eight antigen fragments contained in the Strangvac^®^ vaccine (Intervacc, Hägersten, Sweden), namely SEQ0935 (CNE), SEQ0855 (SclF), SEQ1817 (SclI), SEQ2101 (SclC), SEQ0721 (EAG), SEQ0402 (Eq8), SEQ0256 (Eq5), and SEQ0999 (IdeE), were obtained from available genome data of *S. equi equi* strain Se1866 [31]. This strain was isolated in Sweden in 2000 and was used as antigen source for Strangvac^®^ [31,32,44,45]. Respective DNA sequences were searched in WGS data of the *S. equi equi* isolates of this study using Geneious V.8.1.3. This software was also used to compare these DNA sequences and predicted amino acid sequences in silico with the reference sequences.

### 2.8. Antimicrobial Susceptibility Testing and Assessment for Antimicrobial Resistance Genes

Susceptibility of *S. equi equi* isolates to 15 antimicrobials was assessed by broth microdilution according to Clinical and Laboratory Standards Institute (CLSI) standards. Minimum inhibitory concentrations (MICs) of antimicrobials were determined using Micronaut-S plates for large animals, layout 4 (Code: E1-150-100), in the Micronaut Veterinary System according to the manufacturer’s instructions (Merlin Diagnostika, Bornheim-Hersel, Germany). Horse-derived breakpoints for *S. equi equi*/*S. equi zooepidemicus* were used for ampicillin, ceftiofur, and enrofloxacin, horse-derived breakpoints for streptococci were used for penicillin G, whereas human-derived breakpoints for beta-hemolytic streptococci were used for erythromycin, and tetracycline [46]. MICs for trimethoprim/sulfamethoxazole were interpreted according to a published study [47]. In addition, MIC_50_ and MIC_90_ values were calculated [48].

Acquired antimicrobial resistance (AMR) genes were determined in silico using ResFinder V.4.1 (https://cge.food.dtu.dk/services/ResFinder/, accessed on 22 August 2022) maintained by the Center for Genomic Epidemiology [49]. The selected thresholds were 60% for sequence identity and 60% for coverage length.

## 3. Results

### 3.1. Taxonomic Identification of Streptococcus equi *ssp.* equi

Putative *S. equi equi* isolates were cultured from specimens of all sampled horses. After 24 h of incubation on BAP, single colonies of these bacteria were small (±1–2 mm in diameter), circular, translucent, and mucoid. A broad halo of beta-haemolysis surrounded each colony. Gram staining revealed that the morphology of these bacteria was Gram-positive cocci in chains. According to their enzymatic activities using API^®^ RAPID ID 32 STREP all putative isolates were assigned to *S. equi equi* (99.5% ID). This assignment was confirmed by MALDI-TOF MS. After using the direct transfer protocol for preparation, all isolates scored values greater than 2.00, indicating secure genus *Streptococcus* identification and probable identification of *S. equi equi* according to the scoring scheme provided by the manufacturer (Table 1). When the bacteria were prepared according to the extended direct transfer protocol, five of seven isolates even scored values greater than 2.30, indicating highly probable identification of *S. equi equi* (Table 1). The *sodA* gene of *S. equi* was detected by PCR in all seven *S. equi equi* isolates. Furthermore, each isolate harboured the signature locus ICE*Se2* of *S. equi equi*, and none of them harboured the signature locus ICE*Sz*1 of *S. equi zooepidemicus* (Table 1).

### 3.2. Molecular Typing of Streptococcus equi *ssp.* equi Isolates

**Figure 1:**
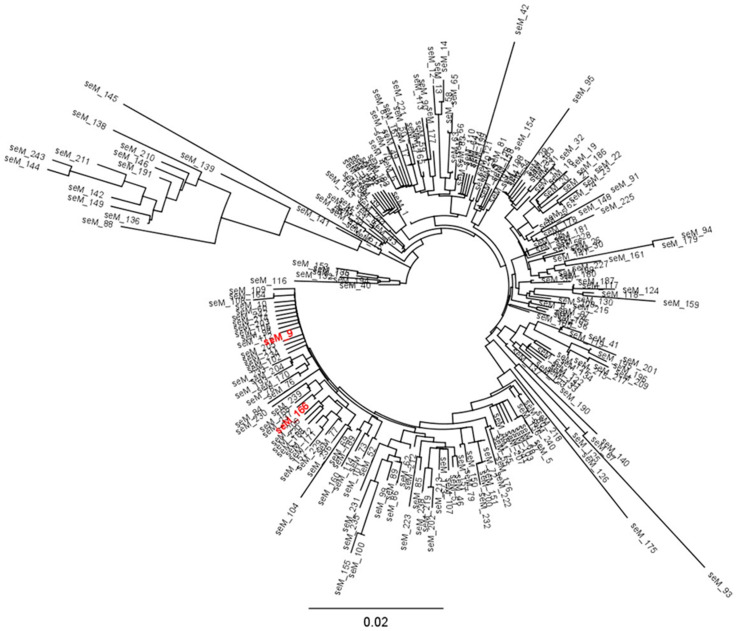
Neighbour-joining phylogenetic tree of *seM* alleles of *Streptococcus equi* ssp. *equi* (n = 244) in the PubMLST database. The scale bar represents the number of substitutions per site in the *seM* variable region (327 bp). Alleles *seM* 166 and *seM* 9 are written in red. The PubMLST [41] was last accessed on 30 September 2022.

**Figure 2:**
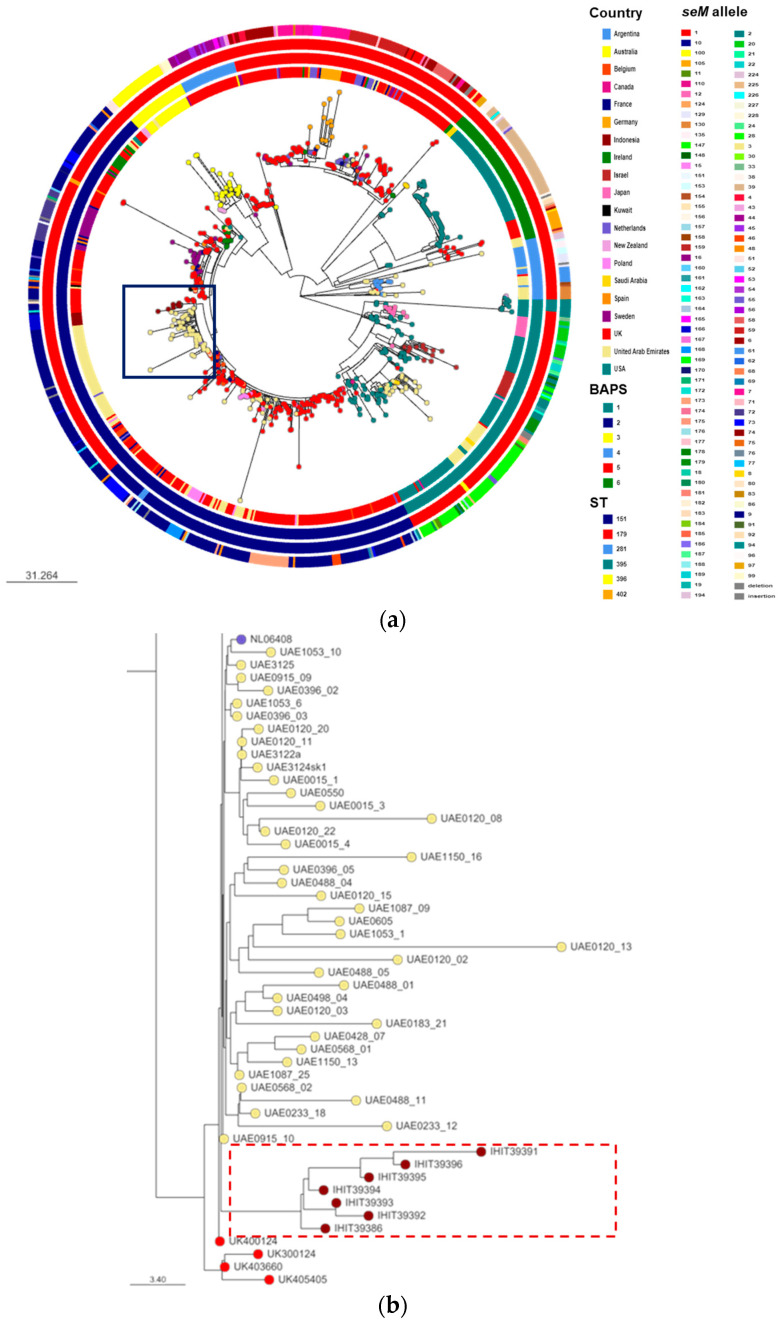
(**a**) Phylogenetic reconstruction of the *Streptococcus equi* ssp. *equi* genomes in this study (n = 7) and publicly available genomes (n = 759) based on pairwise cgMLST scores. Isolates of this study and their closest sub-cluster are indicated in the blue box and visualized in more detail in Figure 2b. (**b**) Phylogentic reconstruction showing that *Streptococcus equi* ssp. *equ*i isolates of this study (red box) built a novel exclusive sub-cluster within Bayesian Analysis of Population Structure (BAPS) cluster 2. The dendrogram was reconstructed from pairwise cgMLST scores using the cgMLST [2] scheme in Pathogenwatch and visualized in Microreact [43]. The scale bars relate to horizontal branch lengths and indicate the number of cgSNPs that are proposed to have occurred on the horizontal branches. Coloured circles indicate the country of origin. The inner to outer circles surrounding the phylogenetic tree represents (1) country of origin, (2) cgMLST BAPS cluster, (3) sequence type (ST), and (4) *seM* allele of each isolate. Colour codes are indicated in the legend.

The seven *S. equi equi* genomes were similar in size, with an average of 2,127,053 bp (min 2,102,418 bp, max 2,187,254 bp). All isolates harboured *seM* allele 166 encoding for SeM peptide 159 (Table 1, Figure 1). MLST revealed that all isolates belonged to ST179 (Table 1, Figure 2).

The cgMLST analysis comprising 1286 core genes of *S. equi equi* revealed that the seven isolates clustered together and belonged to Bayesian Analysis of Population Structure (BAPS) cluster 2 (BAPS-2) (Figure 2a,b). The isolates differed from one another by 2 to 14 pairwise core-genome single-nucleotide polymorphisms (cgSNPs). The number of pairwise cgSNPs between two isolates from the same farms B, C, and D were 2, 12, and 3, respectively. The sub-cluster which had the closest phylogenetic relationship with the *S. equi equi* isolates recovered in Indonesia contained 58 isolates, comprising 55 isolates from horses in the United Arab Emirates (Dubai, 2014), one from the United Kingdom (Cheshire, 2014), one from the Netherlands (unknown location, 2013), and one from Poland (Szamotuly, 2017) (Figure 2b).

### 3.3. Presence and Predicted Amino Acid Sequences of Strangvac^®^ Antigens

In silico analysis revealed that all seven *S. equi equi* isolates from Indonesia harbored the genes encoding for CNE (SEQ0935), SclF (SEQ0855), SclI (SEQ1817), SclC (SEQ2101), EAG (SEQ0721), Eq8 (SEQ0402), Eq5 (SEQ0256), and IdeE (SEQ0999). Furthermore, encoding DNA sequences and predicted amino acid sequences were identical to seven of the eight antigen fragments used for the construction of Strangvac^®^. Regarding Eq8, all *S. equi equi* isolates from Indonesia contained a nonsynonymous nucleotide substitution at position 673 (C → T) of the *eq8* gene, leading to a single amino acid substitution at codon number 225 (H → Y).

### 3.4. Antimicrobial Susceptibility and Resistance Genes of Streptococcus equi *ssp.* equi

All seven *S. equi equi* isolates in this study were invariably susceptible to the tested beta-lactam antibiotics where defined breakpoints were available, namely penicillin G, ampicillin, and ceftiofur. All isolates proved to also be susceptible to tetracycline and erythromycin, but were resistant to enrofloxacin. Five of seven isolates (71.4%) showed intermediate susceptibility or resistance to the combination of trimethoprim and sulfamethoxazole (Table 2). No resistance gene was identified by using ResFinder 4.1 database, which comprised 2690 antimicrobial resistance genes at the time of testing.

## 4. Discussion

Results of the bacteriological examination supported the tentative diagnosis of strangles in the diseased horses from this study. All horses showed typical clinical signs of strangles, including fever, purulent nasal discharge, and enlargement or abscessation in lymph nodes of the head and neck [1,12]. In accordance with these clinical findings, beta-haemolytic streptococci could be isolated from these horses and unambiguously identified as *S. equi equi* by biochemical reactions, enzymatic activities, protein spectra, and presence of marker genes by using API^®^ RAPID ID 32 STREP (bioMérieux), MALDI-TOF MS, and multiplex PCR [35], respectively. The multiplex PCR detected the *sodA* gene encoding the manganese-dependent superoxide dismutase A protein (SodA) [35,50] in all *S. equi equi* isolates. Furthermore, all isolates also harboured the ICE*Se*2 locus. ICE*Se*2 is an integrative conjugative element that is specific for *S. equi equi* and encodes for a non-ribosomal peptide synthetase system [51]. The product of this system, equibactin, is a potential bacterial siderophore that facilitates iron acquisition and may contribute to the virulence of *S. equi equi* [20,51]. In accordance with this observation, all *S. equi equi* isolates from this study displayed a mucoid colony morphotype on BAP, indicating that they produced a capsule and could be considered virulent. Virulent strains of *S. equi equi* almost always have a hyaluronic acid capsule, whereas less virulent strains are non-encapsulated [19,52].

All *S. equi equi* isolates in this study displayed ST179. This result agrees with previous studies confirming that the global population of *S. equi equi* is almost clonal with respect to MLST [2,25,31]. So far, only nine STs of *S. equi equi* have been identified worldwide from 1955 until 2020, namely ST151, ST179, ST281, ST282, ST283, ST325, ST395, ST396, and ST402 (PubMLST database, last accessed 30 September 2022, [2]). All STs are single or double locus variants of ST179, thus belonging to the clonal complex (CC) 179 [2]. Currently, ST179 is the most prevalent ST in the global *S. equi equi* population, followed by its single locus variant ST151 that differs in one nucleotide in the *tdk* gene [2,25]. *S. equi equi* strains of ST179 have highly diverged and today are distributed in all of the cgMLST BAPS clusters [2,32]. Deeper genomic analyses of *S. equi equi* isolates from around the world suggested that international transportation and mixing of horses during the global conflicts in the nineteenth and twentieth century had led to the emergence of a fitter strain of *S. equi equi*, from which all contemporary strains descended [31]. A consequence of this evolution is that typing methods with higher discriminatory power than MLST, such as *seM* typing or cgMLST, are required to break down short-term and small-scale transmissions of strangles.

*SeM* typing, which is based on nucleotide sequence polymorphism in the N-terminal region of the *seM* gene [21], suggested that the same *S. equi equi* strain harbouring *seM* allele 166 had caused the strangles cases investigated in this study. *SeM* allele 166 shares 99.7% nucleotide sequence identity with *seM* allele 9, which is the most prevalent *seM* allele in *S. equi equi* worldwide [2,32]. In fact, both alleles differed by only one nucleotide at position 77 (G → A) in the variable region of the *seM* gene that is used for *seM* typing (327 bp). This nonsynonymous difference at codon number 63 of the *seM* gene resulted in one amino acid difference (arginine → lysine) between the amino acid sequences deduced from *seM* allele 9 and *seM* allele 166, respectively. *SeM* allele 166 has also been detected in two isolates recovered from horses in the Netherlands in 2013 and Poland in 2017, respectively, which both belonged to ST179 and BAPS-2 as well [2]. Discrimination of *S. equi equi* isolates by *seM* typing has been used to trace strangles outbreaks in Europe [21,22,27,53], North America [23,34], South America [54], and Asia [52,55]. Associations between certain *seM* alleles, geography, and history of horse movements can help to enlighten chains of *S. equi equi* infection [27,52]. The number of recognized *seM* alleles is large and still growing. So far, 244 *seM* alleles have been detected worldwide (PubMLST, last accessed 30 September 2022). However, certain *seM* alleles are globally distributed, for example *seM* allele 9 (29.4%, 223 of 759 global isolates) [32], which as a consequence makes it impossible to draw a conclusion about recent transmission when *S. equi equi* strains with this allele are involved. On the other hand, sequence analyses have indicated that the *seM* gene is subject to diversifying selection [21,31] and that its mutation is an ongoing process [27]. Thus, timely identification of different but highly similar *seM* alleles in different horses may not exclude that they belong to the same strangles epizootic [27].

Based on cgMLST, all *S. equi equi* isolates in this study fitted into the current phylogenetic tree of *S. equi equi*. We therefore conclude that the isolates from Indonesia were also descendants of the abovementioned *S. equi equi* strain that has emerged globally since the late nineteenth and early twentieth century. Furthermore, cgMLST analysis revealed that all isolates in this study clustered together as a novel and exclusive subcluster within BAPS-2. Within this subcluster isolates differed only by 2 to 14 pairwise cgSNPs, suggesting that the respective strangles cases were caused by members of the same clonal sublineage of *S. equi equi* and, therefore, were probably epidemiologically linked. Although epidemiological data on the diseased horses were limited, they support the hypothesis that the observed strangles cases in Java, Indonesia, were linked and the causative pathogens were variants of one particular strain of *S. equi equi*. This is obvious for horses 2 and 3, 4 and 5, as well as 6 and 7, respectively, since these were pairs of stablemates and suffered from strangles concurrently. Furthermore, a veterinarian reported retrospectively that horse 1 was transported from farm A (East Java) to farm C (Central Java) after it had recovered from strangles and appeared healthy again. A few weeks later, horses 4 and 5 on farm C fell ill with clinical signs of strangles. The *S. equi equi* isolates from horses 1 and 5 differed from each other by only 2 cgSNPs, thus supporting the assumption that these cases were links in the same infection chain. This finding is in accordance with previous studies showing that, if the guttural pouch is infected, recovered horses can act as carriers and shed *S. equi equi* bacteria via nasal discharge for up to several years [4,8,56]. Interestingly, isolates from horses 4 and 5 (both farm C) were more closely related to isolates from horses 7 (farm D) and 1 (farm A), respectively, than they were to each other. This finding may indicate a mixed infection on farm C with two strains of *S. equi equi* circulating at the same time. Horses 6 and 7 in farm D were infected shortly after a new horse from Central Java had been introduced into the farm. Unfortunately, it could not be clarified whether this new horse from Central Java had any contact with farm C prior to its transport to farm D. Additionally, there were no hints to contacts between farm B and the other farms.

Strangles has been observed in Asian countries before [2], for example, in United Arab Emirates [2], India [57], China [55], and Japan [52]. Particularly in South East Asia, strangles outbreaks have been reported occasionally from Thailand [58], the Philippines [59], Malaysia [60], and Indonesia [28,29]. In Indonesia, *S. equi equi* was previously isolated from a horse in West Java in 2008 [28]. In 2016, district veterinary authorities in Java Island received reports about suspected strangles cases. Surveillance in 2016, 2017, and 2018 confirmed that some horses were serologically positive for *S. equi equi* infection [29]. The total population of horses in Indonesia in 2018 was 421,104, distributed in 34 provinces [61]. There are only horses on Java island, but no donkeys or feral horses [29]. The strangles cases reported in this study occurred in West, Central, and East Java provinces which rank fourth, fifth, and sixth in Indonesia concerning the size of horse population with 13,147, 11,385, and 10,760 horses, respectively [61]. Recently, awareness of horse diseases in Indonesia has increased, particularly as the country hosted the equestrian competitions at the 18th Asian Games in 2018. A surveillance program for equine diseases (including strangles) was conducted in Jakarta and West Java in 2017 and 2018. This surveillance was essential since it was part of the self-declaration of an equine disease-free zone in Jakarta to facilitate horse competitions during the Asian Games event [29]. Although strangles has been listed as a notifiable disease in Indonesia since 2018 [30], to the best of our knowledge, no cases have been officially reported since 2019.

Transportation of clinically healthy carrier horses is a severe factor for spread of *S. equi equi*. Thus, quarantine of newly arrived horses for three weeks with additional screening for carriers are valuable measures to prevent strangles [1]. Quarantine is mandatory in Indonesia for all imported horses, including those imported for international equestrian competitions [62,63,64]. Even so, quarantine at a local level can be challenging due to the frequent movement of horses during breeding and show seasons, particularly in the absence of diagnostic facilities and adequate hygienic measures.

The role of antibiotics in appropriate treatment of horses with strangles is the subject of an ongoing scientific debate [12] since many strangles cases resolve without any application of antimicrobial drugs [65]. In addition, administration of penicillin during acute strangles can interfere with the persistence of humoral immunity to *S. equi equi* [3]. However, abscesses can develop slower or recur if antimicrobial treatment is discontinued [1]. Some authors suggest that antimicrobial treatment can be particularly indicated in certain cases such as acute infection with high fever and depression prior to the formation of abscesses, respiratory distress, profound lymphadenopathy, or metastatic abscessation and guttural pouch infections treated locally and systemically to eliminate the carrier state [1]. Regardless, continuous monitoring of bacterial pathogens for AMR is essential for maximizing the effects of antimicrobial stewardship. In this study, all *S. equi equi* isolates were susceptible in vitro to beta-lactam antibiotics penicillin G, ampicillin, and ceftiofur. These findings are consistent with those of other studies and suggest that penicillin G can also be considered in Indonesia as the drug of first-choice for systemic antibiotic treatment of horses with strangles when it is clinically indicated [1,14,66,67,68]. MIC_90_ of ceftiofur was 0.12 µg/mL in this study, which was the same as in a previous study [69] and which is lower than the CLSI-approved susceptible breakpoint of ≤0.25 µg/mL [46]. In a large outbreak of strangles in the USA ceftiofur showed a greater efficacy than penicillin G and doxycyclin concerning the duration of symptoms [5]. However, ceftiofur, like other third-generation cephalosporins, has been categorized as critically important antimicrobial drug in human medicine [70]. In horses it should be used with restraint and be reserved for cases where compliance to the treatment scheme is difficult to support [1]. Restrained and carefully considered use is also recommended for macrolides such as erythromycin although all *S. equi equi* isolates in this study proved erythromycin susceptible [67,70]. Tetracyclines, such as oxytetracycline and doxycylin, and trimethoprim/sulfonamide combinations are regarded as valuable antimicrobials to treat bacterial infectious diseases in horses [1,66]. In fact, all isolates in this study were susceptible to tetracycline which is in agreement with a previous finding that resistance to this agent is rare in *S. equi equi* [14]. Finding resistance to enrofloxacin and trimethoprim/sulfamethoxazole in all or some isolates from this study, respectively, is also in accordance with previous studies [13,14,15]. Pus inactivates trimethoprim/sulfamethoxazole and makes this drug unsuitable for treating any purulent bacterial infection [67]. Our finding of intermediate or no in vitro susceptibility to trimethoprim/sulfamethoxazole in five of seven *S. equi equi* isolates also argues against its use in horses with strangles. Suitability of the other antimicrobials in this study remains unclear since veterinary breakpoints for MIC interpretation, particularly for *Streptococcus* spp. from horses, are not available [46,47]. Notably, AMR genes were not detected among the *S. equi equi* isolates of this study by using the database of ResFinder 4.1, which comprises over 2690 acquired antimicrobial resistance genes [49].

Despite the availability of some commercial strangles vaccines [1,9,12,45], none is currently available in Indonesia [71]. A novel *S. equi equi* subunit vaccine, termed Strangvac^®^, utilizes a cocktail of distinct peptides of *S. equi equi* as antigens for protective immunization [17,45]. More specifically, Strangvac^®^ contains recombinant internal fragments of eight antigens from *S. equi equi* strain Se1866, namely SEQ0935 (CNE), SEQ0855 (SclF), SEQ1817 (SclI), SEQ2101 (SclC), SEQ0721 (EAG), SEQ0402 (Eq8), SEQ0256 (Eq5), and SEQ0999 (IdeE) [17,32,45]. Since the whole genome sequence of Se1866 is available and vaccine design has been described precisely [45] we took the chance to compare WGS data of the Indonesian *S. equi equi* isolates of this study with sequence data of Strangvac^®^ antigens. In fact, DNA and predicted amino acid sequences of the antigens used in Strangvac^®^, except for the Eq8 fragment, were identical to those of all *S. equi equi* isolates investigated in this study. Regarding the Eq8 antigen, all *S. equi equi* isolates from Indonesia encoded for a protein that carried a tyrosine instead of histidine at position 225. The same substitution was also detected in 99.7% of other BAPS-2 isolates [32], suggesting that this substitution in Eq8 is common to *S. equi equi* strains of BAPS-2. Residue 225 is C-terminal amino acid of the Eq8 antigen fragment used in Strangvac^®^ [32,45]. It was suspected that the terminal amino acid substitution will not significantly affect the antigenicity of this protein relative to Strangvac^®^ [32]. We therefore assume that immunization horses with Strangvac^®^ might also be protective against the *S. equi equi* strains circulating in Indonesia. According to a previous genomic study, the antigen fragments used in Strangvac^®^ are highly conserved in the global *S. equi equi* population [32]. Strangvac^®^ is intended for horses at high risk of infection in areas where *S. equi equi* has been detected [18]. The vaccine has proven to be safe and protective when administered intramuscularly to horses from six months old [17]. A previous study reported that protection increased from 31% to 58% within two weeks and a two months period after second vaccination, respectively, and then increased to 94% within three weeks after third vaccination [17].

## 5. Conclusions

All horses from this study were infected by genetically closely related strains of the same distinct sublineage within BAPS cluster 2 of *S. equi equi*. Although this sublineage is new and only contains *S. equi equi* isolates from Indonesia so far, its members resemble *S. equi equi* strains from other parts of the world concerning in vitro susceptibility to antimicrobials and structure of immunologically important antigens. We therefore suggest that penicillin G can be regarded as first-choice antibiotic in those cases of strangles in Indonesia where antimicrobial treatment is indicated for clinical reasons. In addition, we assume that Strangvac^®^ is also protective in this country when administered to high-risk horses. Further studies are needed to assess the genetic diversity of *S. equi equi* in Indonesia, and examine the possible association of genotypes with provinces, regencies and districts.

## Figures and Tables

**Table 1:** Origin and characteristics of *Streptococcus equi* ssp. *equi* isolates from horses in Indonesia investigated in this study.

Isolate ID	Original Name	Date of Sampling (DD-MM-YYYY)	Horse				Location		MALDI-TOF MS Scores		PCR Results			*seM* Typing		MLST	cgMLST
			No.	Age (Years)	Sex	Specimen	Farm	Province	dt	edt	*sodA*	ICE*Se*2	ICE*Sz*1	Allele	Peptide	ST	BAPS Cluster
IHIT39386	DAR001/18	02-06-2018	1	5	F	PML	A	East Java	2.42	2.43	+	+	-	166	159	179	2
IHIT39393	DAR002/18	21-07-2018	2	4	M	N	B	West Java	2.09	2.28	+	+	-	166	159	179	2
IHIT39392	DAR004/18	21-07-2018	3	5	M	N	B	West Java	2.18	2.35	+	+	-	166	159	179	2
IHIT39391	DAR008/18	09-10-2018	4	8	F	PML	C	Central Java	2.05	2.35	+	+	-	166	159	179	2
IHIT39394	DAR010/18	09-10-2018	5	nd	nd	N	C	Central Java	2.02	2.28	+	+	-	166	159	179	2
IHIT39395	DAR011/18	22-11-2018	6	nd	nd	N	D	West Java	2.42	2.44	+	+	-	166	159	179	2
IHIT39396	DAR012/18	22-11-2018	7	8	nd	N	D	West Java	2.24	2.42	+	+	-	166	159	179	2

Abbreviations: dt, direct transfer protocol; edt, extended direct transfer protocol; ST, sequence type; cgMLST, core genome multilocus sequence typing; BAPS, Bayesian Analysis Population Structure; F, female; M, male; nd, no data available; PML, pus mandibular lymph nodes; N, nasal discharge.

**Table 2:** Antimicrobial susceptibility of *Streptococcus equi* ssp. *equi* isolates from horses in Indonesia (n = 7).

Antimicrobial Substance	Antimicrobial Class	Number of Isolates with the Respective MIC Value (µg/mL)														Percentage (%)			MIC_50_ (µg/mL)	MIC_90_ (µg/mL)
		0.016	0.03	0.06	0.125	0.25	0.5	1	2	4	8	16	32	64	128	**S**	**I**	**R**		
Penicillin G *	Beta-lactams			7	0	0	0	0	0	0	0					100	0	0	≤0.0625	≤0.0625
Amoxicillin/Clavulanic acid	Beta-lactams								6	1	0	0				-	-	-	≤2/1	4
Ampicillin **	Beta-lactams					7	0	0	0	0	0	0				100	0	0	≤0.25	≤0.25
Ceftiofur **	Beta-lactams				7	0	0	0	0	0						100	0	0	≤0.125	≤0.125
Cephalothin	Beta-lactams							7	0	0	0	0				-	-	-	≤1	≤1
Enrofloxacin **	Fluoroquinolons	0	0	0	0	0	1	6								0	0	100	1	1
Florfenicol	Phenicols							4	3	0	0					-	-	-	≤1	2
Gentamicin	Aminoglycosides				0	0	0	0	0	5	2					-	-	-	4	8
Spectinomycin	Aminoglycosides									0	0	0	0	3	4	-	-	-	>64	>64
Trimethoprim/Sulfa-methoxazole ****	Folate pathways inhibitors					0	2	4	0	1						28.6	57.1	14.3	1/19	>2/38
Tetracycline ***	Tetracyclines				5	1	1	0	0	0	0					100	0	0	≤0.125	0.5
Tiamulin	Pleuromutilins					0	0	0	7	0	0	0	0			-	-	-	2	2
Erythromycin ***	Macrolides				7	0	0	0	0	0						100	-	-	≤0.125	≤0.125
Tilmicosin	Macrolides						0	5	1	1	0	0				-	-	-	1	4
Tulathromycin	Macrolides							0	0	4	3	0	0	0		-	-	-	4	8

* horse-derived breakpoints for streptococci available [46], ** horse-derived breakpoints for *S. equi equi* and *S. equi zooepidemicus* available from CLSI (2020) [46], *** human-derived breakpoints for streptococci available [46], **** horse-derived breakpoints for *S. equi equi* and *S. equi zooepidemicus* according to Sadaka et al. (2017) [47]. (S) susceptible, (R) resistant, (I) intermediate. White areas indicate the dilution ranges tested. Numbers shown above this range represent isolates with MICs greater than or equal to the concentration shown. Numbers at the lower end of dilution ranges represent isolates with MICs equal or lower than the lowest concentration tested. Where available, breakpoints are indicated by the vertical line (susceptible—intermediate; I) and/or the double vertical line (susceptible or intermediate—resistant; R). Grey shaded areas indicate concentrations of antimicrobials that were not tested. Amoxicillin/clavulanic acid (2:1); trimethoprim/sulfamethoxazole (1:19). MICs were determined by broth microdilution susceptibility testing as recommended by CLSI [46].

## Data Availability

Genomes and metadata presented in this study have been deposited in Pathogenwatch and can be accessed at https://pathogen.watch/collection/pi6lq3wwlwkw-paper-2022-see-indonesia-766-genomes. Visualization of the metadata and phylogenetic reconstruction can be accessed at https://microreact.org/project/bW2dCd66hx3A9wxbGT1rW4-s-equi-equi-indonesia-7-and-worldwide-759.
